# Fatal pediatric case of Kounis syndrome and sepsis: a case report

**DOI:** 10.1186/s12245-025-00886-4

**Published:** 2025-05-01

**Authors:** Tamara Berezna, Olha Synoverska, Nadiya Fomenko, Iryna Pylyuk, Khrystyna Lazurkevych, Viktoria Bedei, Taras Kotyk

**Affiliations:** 1 Department of Children’s Diseases of Postgraduate Education, Ivano-Frankivsk National Medical University, Halytska 2, Ivano-Frankivsk, 76018 Ukraine; 2 Division of Pulmonology, Center of Infection Diseases, Ivano-Frankivsk, 76018 Ukraine; 3 Department of Human Anatomy, Ivano-Frankivsk National Medical University, Halytska 2, Ivano-Frankivsk, 76018 Ukraine

**Keywords:** Case report, Children, Kounis syndrome, Sepsis

## Abstract

**Background:**

Kounis syndrome is a hypersensitivity reaction that induces acute coronary artery events, nevertheless its pediatric occurrence remains rare and often underdiagnosed. This report describes a fatal case of Kounis syndrome triggered by ceftriaxone-lidocaine administration in a child, in the context of sepsis and multiple organ dysfunction syndrome.

**Case presentation:**

A 4-year girl with a history of cyclic vomiting syndrome, was admitted to the ICU with severe lethargy and pallor 30 min after the second intramuscular injection of ceftriaxone, which had been prescribed for vomiting, diarrhea, and fever. Her laboratories were pertinent for a metabolic acidosis, neutrophilic leukocytosis, renal dysfunction, elevated cardiac markers (troponin I and cardiac-type creatine phosphokinase), EKG signs of myocardial ischemia, bilateral bronchopneumonia, and right lower multifocal pneumonia. Despite intensive management, the patient’s condition continued to deteriorated, which lead to multiple organ dysfunction and eventual death.

**Conclusion:**

This case highlights the need for heightened clinical awareness of Kounis syndrome in pediatric settings, especially in patients with underlying infections. This case underscores the fatal potential of undiagnosed Kounis syndrome in the pediatric population and highlights the urgent need for enhanced vigilance and multidisciplinary preparedness.

## Introduction

Kounis syndrome is a hypersensitivity reaction triggered by allergy or anaphylaxis, potentially leading to acute coronary syndrome [1]. The first clinical case of Kounis syndrome was described in 1950 and the first pediatric case was reported in 2009 [2]. Despite increasing medical attention, Kounis syndrome remains underreported and underdiagnosed, as it is considered to be a rare and challenging diagnosis [3]. Herein, we present a fatal case of Kounis syndrome in a child with sepsis, infectious-toxic shock, and multiple organ dysfunction syndrome.

## Case report

### Patient information

A 4-year-old girl was urgently admitted to the ICU of the Regional Children’s Clinical Hospital at 2^40^ a.m.

#### Signs and symptoms

Severe lethargy, pallor, and delayed responsiveness, which developed 30 min after the second intramuscular injection of ceftriaxone with lidocaine.

#### Clinical course before admission

Her parents denied any medical history of respiratory or other infections within 3 weeks before her admission. The illness developed acutely and was characterized by fever, vomiting, and diarrhea. There were no noted skin manifestations. Due to the patient developing these acute symptoms, her parents were referred to the primary health care unit for treatment of a likely bacterial infection. Considering the severity of the patient’s condition and the absence of bacteriological test results and antibiotic susceptibility data, ceftriaxone was empirically chosen as a broad-spectrum antibiotic for management of her likely bacterial infection.

#### Medical and family history

The parents denied a history of any significant medical conditions except for cyclic vomiting syndrome, which occurred several times over the past year. Her family history was pertinent only for type 2 diabetes mellitus. The parents denied any known allergies and she was up to date on routine vaccinations for her age.

### Timeline

**Table:** 

Date/Time	Event Description
Day 1	Onset of fever, vomiting, and diarrhea
Day 2	Initial hospital admission with suspected infection intestinal disease; starting management with ceftriaxone
Day 2 (9:00 pm)	Development of allergic reaction (30 min after the second dose of ceftriaxone)
Day 3 (2:40 am)	Transferred to Regional Children’s Hospital ICU
Day 3 (09:00 am)	Worsening condition, placed on mechanical ventilation, pharmacologic coma induced
Day 3 (1:30 pm)	Progressive decline in ejection fraction to 15%
Day 3 (8:00 pm)	Progressive deteriorating coagulation parameters
Day 3 (10:10 pm)	Asystole occurred
Day 3 (10:40 pm)	Death of the patient

### Clinical findings

The patient was a well-nourished girl with a body weight of 20 kg (+ 1σ). Vital signs included a body temperature of 36.5 °C and saturation (SpO₂) between 96% and 88%. The skin was markedly pale and dry and exhibited reduced turgor with perioral cyanosis. The child was stuporous (Glasgow Coma Scale score of 12 and a white spot test > 5 s), tachypnenic with a respiratory rate of 24 breaths per minute. On auscultation, there was diminished breath sounds, scattered moist rales, dullness in the lower lung fields bilaterally. She was tachycardic with a heart rate of 138 bpm, and on cardiac auscultation she had weak heart sounds. Her blood pressure on admission was 140/100 mmHg, nevertheless, it decreased to 75/35 mmHg. There was no organomegaly on abdominal exam with the liver palpable 1.5 cm below the costal margin. Unfortunately, the spleen was not palpable during the physical exam. The patient had been anuric for the past 24 h. The diarrhea (7–8 times daily) over the last 48 h, which coincided with the onset of fever and vomiting.

### Diagnostic assessment

SARS-CoV-2 antigen rapid test: negative.

Laboratory analysis revealed anemia, neutrophilic leukocytosis, and elevated inflammatory markers. In addition there was a noted metabolic acidosis, hypoproteinemia, renal dysfunction, elevated liver enzymes, and hypoglycemia. Cardiac biomarkers and D-dimer levels were markedly elevated. All laboratory findings can be seen in Table 1. Coagulation parameters at two-time points are summarized in Table 2.

**Table 1:** Laboratory findings

	Value	Reference range
Complete Blood Count		
Hemoglobin (Hb), g/L	96	110–175
Erythrocytes (RBCs), × 10¹²/L	3.4	3.5–5.6
Mean Corpuscular Volume (MCV), µm^3^	86.7	70–110
Hematocrit (Hct), %	29.31	32–44
Leukocytes (WBCs), × 10⁹/L	30.9	4.5–13.5
Myelocytes	4	0
Metamyelocytes	5	0
Bands (p), %	37	1.0–6.0
Segmented neutrophils (s), %	43	47–72
Eosinophils, %	0	0.5-5.0
Lymphocytes, %	5	22.0–76.0
Monocytes, %	6	2.0–12.0
Platelets, × 10⁹/L	178	160–400
Erythrocyte Sedimentation Rate (ESR), mm/h	62	4–15
**Biochemical Blood Panel**		
Glucose, mmol/L	1.7	4.2–6.4
Total Protein, g/L	48.7	52–76
Serum Uric Acid, mmol/L	17.9	1.8–6.5
Creatinine, µmol/L	237	27–62
Alanine Aminotransferase (ALT), U/L	89	0-39.9
Aspartate Aminotransferase (AST), U/L	244	0-39.9
**Electrolyte Balance**:		
Potassium (K⁺), mmol/L	3.77	3.4–4.7
Sodium (Na⁺), mmol/L	141	138–145
Chloride (Cl⁻), mmol/L	103.8	95–110
**Arterial Blood Gas (ABG) Analysis**:		
pH	7.02	7.35–7.45
pCO₂, mmHg	21	34–45
pO₂, mmHg	91	75–100
HCO₃⁻, mmol/L	5.4	22–26
Base Excess, mmol/L	24.0	-2_+2
**Inflammatory and Cardiac Biomarkers**		
Procalcitonin, ng/mL	15.25	0-0.5
D-Dimers, ng/mL	2170.4	0-250.0
C-reactive protein (CRP), mg/L	48	0-5.9
Serum Troponin I, ng/mL	2.95	0-0.5
Cardiac-type Creatine Phosphokinase (CK-MB), U/L	75	0-4.9

**Table 2:** Coagulation parameters at different time points

	2^00^a.m.	8^00^p.m.
Prothrombin time	15.8 s	60.9 s
Prothrombin index	179.4%	158.9%
Fibrinogen	2.34 g/l	2.8 g/l
International Normalized Ratio	1.33	5.41
Activated Partial Thromboplastin Time	36.3 s	46.7 s

Upon admission to the ICU of the Regional Children’s Clinical Hospital, stool, urine, and blood samples were collected for microbiological testing. Nevertheless, these were non-revealing as the results were negative, with no microorganisms identified.

**Fig. 1:**
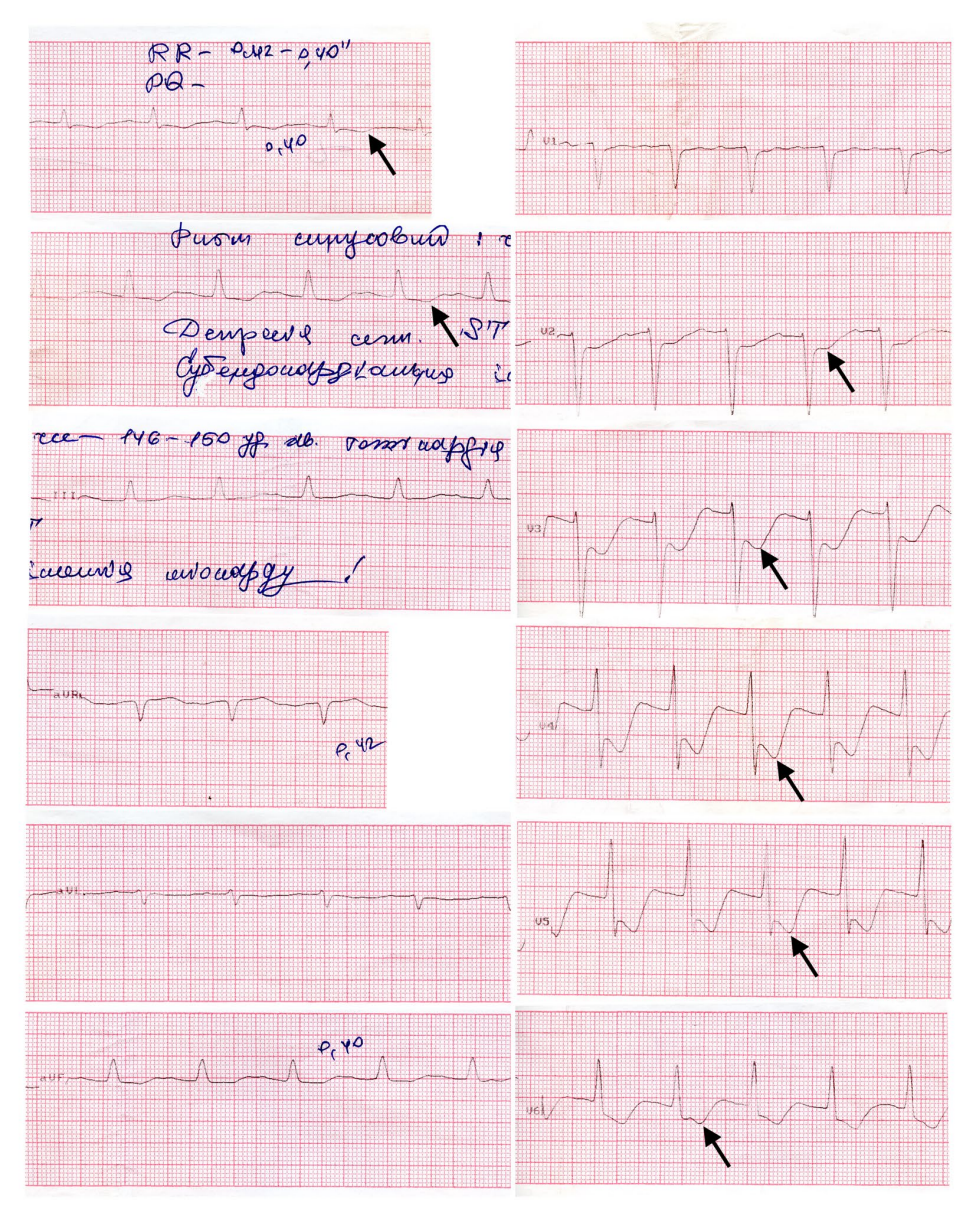
The ECG findings with signs of subendocardial myocardial ischemia. Arrows indicate ST-segment depression

Electrocardiography (ECG) revealed sinus tachycardia with clinically significant ST-segment depression (Fig. 1).

**Table 3:** Echocardiographic findings at different time points

	9^00^a.m.	1^30^p.m.	4^00^p.m.
Ejection Fraction (EF, %)	30	15	28
Left Ventricular End-Diastolic Diameter (LVEDD, mm)	33	35	34
Left Ventricular End-Diastolic Volume (LVEDV, mL)	34	32	32
Mitral Valve Insufficiency	mild	moderate	moderate
Mitral Valve Regurgitation	+	++	++
Tricuspid Valve Insufficiency	mild	moderate	moderate
Tricuspid Valve Regurgitation	+	++	++

Echocardiography revealed significant cardiac impairment (Table 3). Moderate mitral and tricuspid valve insufficiency were present, but myocardial wall thickness remained normal. No coronary artery abnormalities were observed.

Chest X-ray: bilateral bronchopneumonia and multifocal pneumonia in the right lower lobe.

Ultrasonography revealed renal parenchyma with markedly increased echogenicity and reduced systolic flow, no other significant changes were noted.

### Therapeutic intervention

Upon admission, the patient’s condition worsened due to multiple organ dysfunction syndrome, hemodynamic instability, and toxicosis (Phoenix Sepsis Score − 8). At 9:00 a.m., the child was placed on mechanical ventilation and induced into a pharmacologic coma (dobutamine 5–20 µg/kg/min, norepinephrine 0.2 µg/kg/min, dopamine 10 µg/kg/min, morphine 0.2 µg/kg). The treatment regimen included dobutamine, norepinephrine, dopamine, levosimendan, hydrocortisone, dexamethasone, chlorpyramine, morphine, diazepam, and meropenem. At 10:10 p.m., asystole was observed, and despite resuscitation efforts, the death occurred at 10:40 p.m.

### Outcomes

**Fig. 2:**
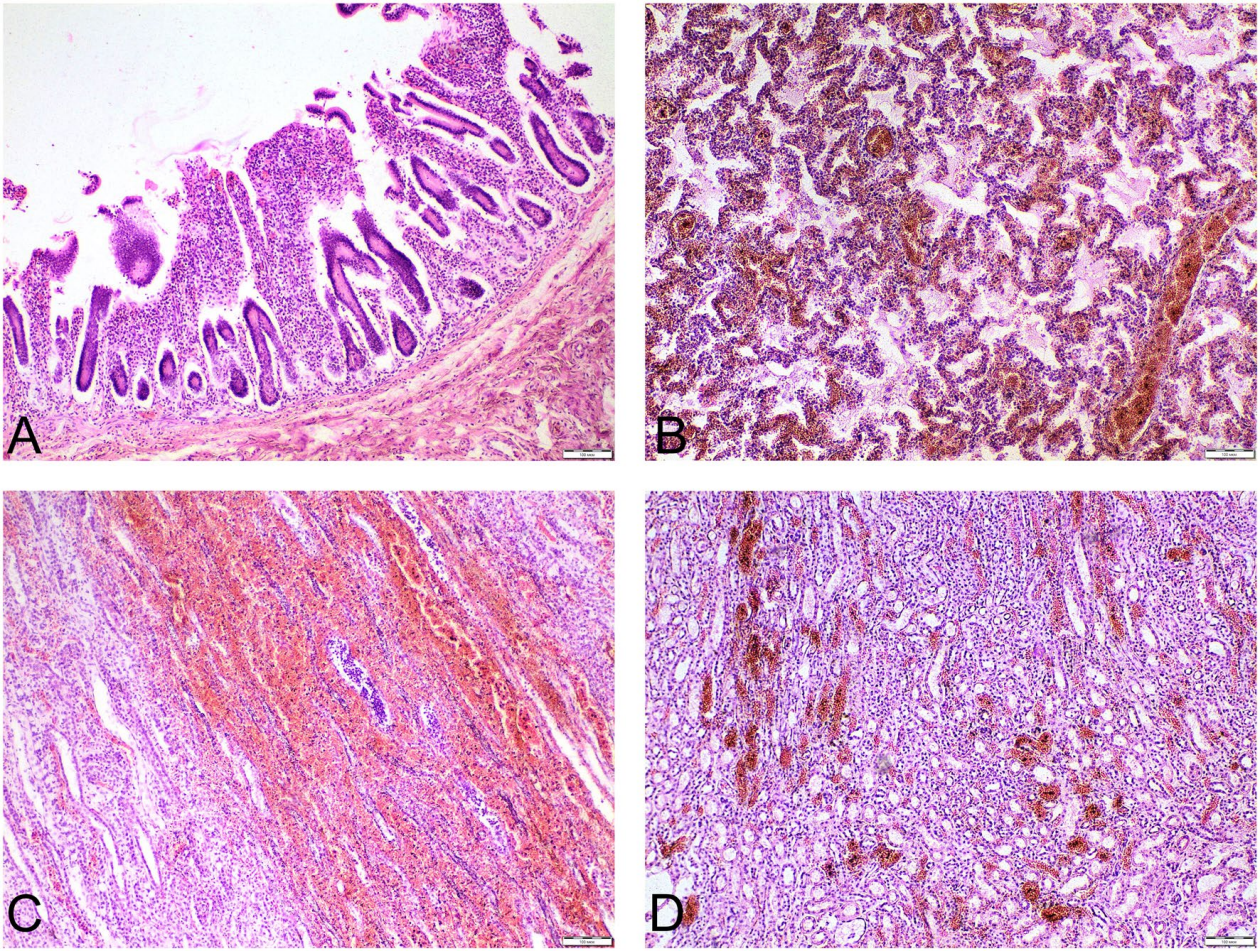
The histopathological findings. **A** – Fragment of jejunum with edematous mucosa and neutrophilic inflammatory infiltrates. Desquamation of the epithelium with a tendency toward erosive changes, signs of active crypt epithelial regeneration, and occasional crypt abscesses are observed (acute bacterial enteritis). **B** – Lung tissue with thickened interalveolar septa and mononuclear inflammatory infiltrates with admixture of neutrophils. The alveolar epithelium is flattened and damaged; numerous hyaline membranes are observed. Occasional alveolar spaces contain exudate. Vascular congestion and microthrombosis (pneumonia-associated diffuse alveolar damage). **C** – Adrenal gland with massive extravasation of erythrocytes (hemorrhage) into the cortical and medullary regions, causing disruption of the gland’s histoarchitecture. Microloci of coagulative necrosis with loss of cellular borders and karyolysis. Microloci of inflammatory reaction zones (Waterhouse-Friderichsen syndrome). **D** – Renal medulla with mostly preserved histoarchitecture. Foci of tubules show necrotic and apoptotic epithelial changes. Numerous vessels exhibit signs of stasis and thrombosis, with single perivascular inflammatory cells present (focal acute tubular necrosis of renal tubular epithelium, signs of thrombovasculitis)

The pathological autopsy report: subtotal bacterial necrotizing pneumonia, complicated by sepsis, which manifested as bacterial thrombovasculitis affecting the intrapulmonary, splenic, mesenteric and renal vessels, along with infectious-toxic shock, severe circulatory disturbances, and dystrophic changes. Additional findings included Waterhouse-Friderichsen syndrome, cerebral edema with brain swelling, leukomalacia, pulmonary edema, and vacuolar degeneration of hepatocytes, cardiomyocytes, and renal tubular epithelium, all against a background of thymic hypoplasia. The key findings are presented in Fig. 2.

The final clinical diagnosis: Bilateral focal necrotizing pneumonia with abscess formation, complicated by toxic syndrome and grade III respiratory failure; infectious-toxic shock; sepsis with multiple organ dysfunction syndrome; anaphylactic shock; and Kounis syndrome, type I.

## Discussion

Kounis syndrome remains underdiagnosed globally due to limited awareness among clinicians. Despite an incidence of 19.4 per 100,000 hospital admissions and a low fatality rate (0.0001%), it is frequently overlooked in clinical practice [4, 5]. While predominantly observed in adults, its incidence in children is six times lower (3.3 per 100,000 inhabitants) [6].

Diagnosis relies on clinical presentation, ECG and echocardiography findings, and laboratory markers [7]. Symptoms often include allergic reactions with variable ECG changes [4], making laboratory tests (e.g., cardiac enzymes, troponins) crucial for confirmation [8]. However, the case with clinical manifestation of anaphylaxis and ECG signs (ST elevation) without significant laboratory findings (normal troponin level and coagulation parameters) is reported [9], additionally highlighting diagnostic challenges. In our case, the second dose of ceftriaxone-lidocaine administration led to cardiovascular compromise within 30 min (subendocardial ischemia, elevated serum troponin I and CK-MB levels), meeting criteria of anaphylaxis according to [10]. According to a systematic review of pediatric patients with Kounis syndrome [11], ST elevation is far more common (63.6%) than ST depression (18.2%). Symptom onset varies significantly (from less than 30 min to 4–6 h after exposure to triggers), with elevated serum troponin I observed in 81.8% of cases and increased CK-MB levels in 63.6%, findings that align with our case. Youcefi HE et al. noted ECG ischemia changes in 95% of cases and elevated cardiac enzymes in 85% [12]. However, a decreased EF (< 50%), as observed in our case, was reported in only 25% of pediatric cases [12].

The fatal outcome in this case resulted from a combination of Kounis syndrome and sepsis with multiple organ dysfunction syndrome. However, at the time of initial hospital admission, the patient presented only with fever, diarrhea, and vomiting, without any other complaints or signs. Due to the circumstances of illness onset (acute onset at a holiday destination) and the lack of advanced healthcare opportunities at the local primary healthcare unit, empirical antibiotic treatment was initiated. Hosoda T et al. described a case of enterocolitis with SARS-CoV-2 excretion without any respiratory symptoms or fever but with a negative SARS-CoV-2 PCR test on throat swabs [13]. Vendargon S et al. documented three pediatric cases where patients initially presented with abdominal pain, but pneumonia was later diagnosed [14]. In one case, microbiological testing was negative, Gram-negative bacilli were isolated from pus in another, and Staphylococcus aureus was identified in the third. Similarly, Naccour J et al. described a 44-year-old male admitted with abdominal pain and vomiting, without respiratory complaints; however, auscultation a few days later revealed right basal crackles, and pneumonia was confirmed by chest X-ray [15]. Zeng S et al. reported an episode of acute hemorrhagic necrotizing enteritis progressing to bilateral pneumonia with consolidation, and severe sepsis and septic shock [16]. However, the patient’s condition deteriorated rapidly due to the development of sepsis and multiorgan dysfunction syndrome, including respiratory failure, leading to oxidative stress imbalance and the progression of metabolic acidosis [17, 18]. In addition, concurrently a hypersensitivity reaction culminated in acute coronary syndrome, resulting in a fatal outcome within less than a day.

The triggers of Kounis syndrome are diverse and include medical drugs, food, vaccines, and insect stings. Among drugs, antibiotics are frequent triggers, particularly in adults [4, 12], including but not limited by amoxicillin, ampicillin, amikacin, ceftriaxone, cephalosporin, cephazolin, cefoxitin, cefuroxime, cephaloridine, cinoxacin, lincomycin, penicillin, ampicillin/sulbactam, sulbactam/cefoperazone, piperacillin/tazobactam, trimethoprim-sulfamethaxazole, sulperazon, vancomycin [4, 12, 19–22]. In pediatric cases, Kounis syndrome is most commonly associated with amoxicillin/clavulanic acid [11, 12], with no documented cases linked to cephalosporins having been reported. While the likelihood of cross-reactivity between penicillins and cephalosporins is considered negligible [23, 24], it is not entirely impossible. Therefore, prescribing antibiotics to patients should be approached with caution due to the potential risk of an allergic reaction and Kounis syndrome development. In the presented clinical episode, intramuscular injection of ceftriaxone-lidocaine led to Kounis syndrome. On the other hand, local anesthetics may also act as triggers, as reported in adult case studies [12, 25, 26]. However, despite the lack of reported cases in the pediatric population, their role in inducing Kounis syndrome cannot be excluded [12].

## Conclusion

This case highlights the diagnostic challenges of Kounis syndrome in pediatric patients, particularly along with severe infectious complications. The fatal outcome underscores the importance of considering allergic myocardial involvement in critically ill children with signs of cardiac ischemia following drug administration. Increased awareness and early recognition of Kounis syndrome could improve outcomes through timely intervention and tailored therapeutic strategies.

## Data Availability

No datasets were generated or analysed during the current study.
